# Effect of ivermectin on the larvae of *Anopheles gambiae* and *Culex quinquefasciatus*

**DOI:** 10.1186/s13071-016-1417-5

**Published:** 2016-03-08

**Authors:** Yahya A. Derua, Bernard B. Malongo, Paul E. Simonsen

**Affiliations:** National Institute for Medical Research, Amani Research Centre, P. O. Box 81, Muheza, Tanga Tanzania; Department of Veterinary Disease Biology, Faculty of Health and Medical Sciences, University of Copenhagen, Dyrlægevej 100, 1870 Frederiksberg C, Copenhagen, Denmark

**Keywords:** Ivermectin, Mosquito larvae, *Anopheles gambiae*, *Culex quinquefasciatus*

## Abstract

**Background:**

Ivermectin is used extensively globally for treatment of helminthic and ectoparasitic infections in animals and humans. The effect of excreted ivermectin on non-target organisms in aquatic and terrestrial environments has been increasingly reported. Due to its low water solubility and adsorption to sediments, the ivermectin exposure-risk to aquatic organisms dwelling in different strata of water bodies varies. This study assessed the survival of larvae of *Anopheles gambiae* Giles and *Culex quinquefasciatus* Say, when exposed to low concentrations of ivermectin under laboratory conditions.

**Methods:**

A total of 1800 laboratory reared mosquito larvae of each species were used in the bioassays. Twelve replicates were performed, each testing 6 concentrations of ivermectin (0.0, 0.001, 0.01, 0.1, 1.0 and 10.0 parts per million (ppm)) against third instar larvae of *An. gambiae* and *Cx. quinquefasciatus*. Larval mortality was recorded at 24 and 48 h post addition of ivermectin.

**Results:**

Survival declined markedly with increase in ivermectin concentration in both species. While mean survival of *An. gambiae* at 24 h of exposure was 99.6 %, 99.2 % and 61.6 % in 0.001, 0.01 and 0.1 ppm of ivermectin, respectively, the mean survival of *Cx. quinquefasciatus* at the same dosage and time was 89.2 %, 47.2 % and 0.0 %. A similar pattern, but with higher mortality, was observed after 48 h of exposure. Comparison between the species revealed that *Cx. quinquefasciatus* larvae were significantly more affected by ivermectin than those of *An. gambiae*, both at 24 and 48 h*.*

**Conclusions:**

Low concentrations of ivermectin in the aquatic environment reduced the survival of larvae of *An. gambiae* and *Cx. quinquefasciatus*, with the effect being more marked in the latter species. It is suggested that this difference may be due to the different water strata occupied by the two species, with ivermectin adsorbed in food that sediment being more readily available to the bottom feeding *Cx. quinquefasciatus* than the surface feeding *An. gambiae* larvae.

## Background

Ivermectin is an important drug for treatment of many helminthic and ectoparasitic infections in animals and humans globally [1]. It has been extensively used in the veterinary field, and its use in humans has recently been scaled up in large programmes to control lymphatic filariasis and onchocerciasis in endemic areas particularly in sub-Saharan Africa [2, 3]. Due to its effectiveness in treating these infections, many of which are particularly common in the tropics, it has been referred to as a ‘wonder drug’ [4]. However, in addition to the anti-parasitic potentials, ivermectin has a broad spectrum of activity against a wide range of other invertebrates, and its effect in the environment on non-target aquatic and terrestrial organisms has been increasingly documented [5].

In relation to its broad spectrum of activity, the ongoing Global Programme to Eliminate Lymphatic Filariasis has reported promising beyond-programme benefits of ivermectin mass drug administration (MDA), including simultaneous curative effects on intestinal and skin parasitic infections [3]. Entomological field studies in the programme areas have moreover documented a decline in survival of female *Anopheles gambiae* that feed on humans shortly after mass treatment with ivermectin [6–8]. Laboratory studies have confirmed this effect of ivermectin on adult anopheline vector survival, and have also demonstrated a reduced fecundity of these vectors following a blood meal from ivermectin treated humans or cattle [9, 10]. Due to this effect on important malaria vectors in sub-Saharan Africa, ivermectin has been considered as a potential tool for future malaria control [11, 12].

Other studies have demonstrated that non-target organisms in terrestrial environments can be affected by faecal excreta from ivermectin-treated animals, while in aquatic environments they can be affected both directly from excreta dropped in water or indirectly through runoff of ivermectin contaminants [13, 14]. Extensive literature on the effect of ivermectin on non-target organisms living in terrestrial and aquatic environments has been summarized previously [5]. Ivermectin has low water solubility and partitions rapidly in aquatic environments from the water phase to sediment particles [15–17]. Its low water solubility and rapid adsorption to sediments suggest that ivermectin may pose a variable risk of exposure to aquatic organisms living or feeding in different strata of the water body.

In sub-Saharan Africa, *An. gambiae* is the most important vector of malaria, and this species, as well as *Culex quinquefasciatus,* are important vectors of lymphatic filariasis. It is possible that the widespread use of MDA with ivermectin could have affected transmission of these infections through an effect on the mosquito vector larvae, and perhaps even could have contributed to the marked change in composition of the vector mosquitoes observed in recent years in north eastern Tanzania [18, 19]. In their aquatic habitat anopheline and culicine larvae feed preferentially in different strata of water but both come to the surface for breathing. While anopheline larvae are mainly surface feeders, culicine larvae feed in the sediment at the bottom of water strata [20]. This feeding behavior may most likely lead to differential ivermectin exposure-risks between the two mosquito species if they reside in ivermectin contaminated habitats. Studies have shown that different species of mosquito larvae are susceptible to low concentrations of ivermectin in laboratory settings as well as in natural mosquito breeding habitats [21, 22]. On this background, the present study assessed and compared the survival of *An. gambiae* and *Cx. quinquefasciatus* mosquito larvae when exposed to low concentrations of ivermectin under laboratory conditions.

## Methods

### Mosquito larvae

Larvae of *An. gambiae* sensu stricto (a colony originating from Kisumu, Kenya) and *Culex quinquefasciatus* (a colony originating from Arusha, Tanzania) mosquitoes were used for the laboratory bioassays. Both colonies had been maintained for several generations at the insectary of the National Institute for Medical Research, Amani Research Centre, Tanga, Tanzania, and were previously also used in a study on the effect of human ivermectin treatment on blood feeding adult mosquitoes [10]. They were maintained using recommended standard mosquito rearing techniques [23]. Larvae of *An. gambiae* and *Cx. quinquefasciatus* were fed with Nutrafin® fish food (Hagen, Taiwan) and Whiskas® cat food (Mars Africa, South Africa), respectively before and during bioassays. Although both foods initially float on the surface of the water, the cat food sinks to the bottom relatively quickly compared to the fish food.

### Preparation of ivermectin solutions

An ivermectin stock solution was first prepared by dissolving 200 mg powdered ivermectin (Sigma-Aldrich, St. Louis, USA) in 20 ml dimethyl sulphoxide (DMSO) (Hybri-Max®, Sigma-Aldrich, St. Louis, USA). The resultant 1.0 % (10 mg/ml) stock solution was kept frozen in 2 ml aliquots until use. On the experimental day, one aliquot of stock solution was thawed and serially diluted in distilled water as previously recommended [24]. In brief, a ten-fold dilution series was prepared by first transferring 2 ml of stock solution to 18 ml of distilled water to make a 0.1 % concentration, and by subsequently repeating this procedure by transferring 2 ml of the latest solution to 18 ml of distilled water to make 0.01, 0.001, 0.0001 and 0.00001 % concentrations of ivermectin. Control solutions (with no ivermectin) comprised of 2 ml DMSO in 18 ml distilled water.

### Larval bioassays

Bioassays with *An. gambiae* and *Cx. quinquefasciatus* larvae were run simultaneously (in parallel). For each species twelve replicates were performed, with four replicates starting on three separate dates. In each experiment, 6 test cups with mosquito larvae were exposed to six different concentrations of ivermectin (including the negative control). At the beginning of experiments, 25 third instar larvae were transferred from the larvae rearing pans to the labeled disposable plastic test cups with 100 ml of filtered non-chlorinated tap water by use of disposable Pasteur pipettes. By using a pipette with disposable tips, and starting with the lowest concentration, 1 ml of each of the six concentrations of ivermectin solutions were then added to the experimental cups (with mosquito larvae) thus giving final concentrations of 0.0, 0.001, 0.01, 0.1, 1.0 and 10.0 parts per million (ppm; equivalent to mg/liter), respectively. The test cups were held at 28 °C and photoperiods of 12 h light followed by 12 h darkness. Test larvae were provided with larval food at onset of each experiment. Larval mortality was recorded at 24 and 48 h after the addition of ivermectin solutions.

### Data analysis

Data were entered in Excel and subsequently analyzed in IBM SPSS Statistics (version 22). Survival of *An. gambiae* and *Cx. quinquefasciatus* was compared using Student’s *t*-test and *P*-values < 0.05 were considered statistically significant.

### Ethics

The study received ethical approval from the Medical Research Coordinating Committee of the National Institute for Medical Research, Tanzania (Ref: NIMR/HQ/R.8a/Vol. IX/1554).

## Results

Twelve replicates, each testing 6 concentrations of ivermectin (including the negative control) each with 25 larvae were conducted in parallel for a total of 1800 larvae of both *An. gambiae* and *Cx. quinquefasciatus*. The mean numbers (and range) of the two species surviving at the different ivermectin concentrations at 24 and 48 h post exposure is shown in Table 1.

**Table 1:** Survival of *An. gambiae* and *Cx. quinquefasciatus* larvae at different ivermectin concentrations and exposure times

Ivermectin concentration^b^	Mean no. ± SD (range) surviving at 24 hours^a^			Mean no. ± SD (range) surviving at 48 hours^a^		
	*An. gambiae*	*Cx. quinquefasciatus*	*p*-value*	*An. gambiae*	*Cx. quinquefasciatus*	*p*-value*
0.0	25.0 ± 0.0 (25–25)	25.0 ± 0.0 (25–25)	NS	24.9 ± 0.3 (24–25)	24.1 ± 1.3 (21–25)	NS
0.001	24.9 ± 0.3 (24–25)	22.3 ± 2.5 (17–25)	0.002	23.7 ± 1.3 (21–25)	19.3 ± 4.4 (12–25)	0.003
0.01	24.8 ± 0.5 (24–25)	11.8 ± 5.3 (4–22)	<0.001	20.2 ± 2.5 (15–23)	4.5 ± 3.9 (0–12)	<0.001
0.1	15.4 ± 6.3 (3–23)	0.0 (0–0)	-	0.08 ± 0.3 (0–1)	0.0 (0–0)	-
1.0	0.0 (0–0)	0.0 (0–0)	-	0.0 (0–0)	0.0 (0–0)	-
10.0	0.0 (0–0)	0.0 (0–0)	-	0.0 (0–0)	0.0 (0–0)	-

^a^Twelve replicates each with 25 larvae

^b^In parts per million

*t-test

**Fig. 1:**
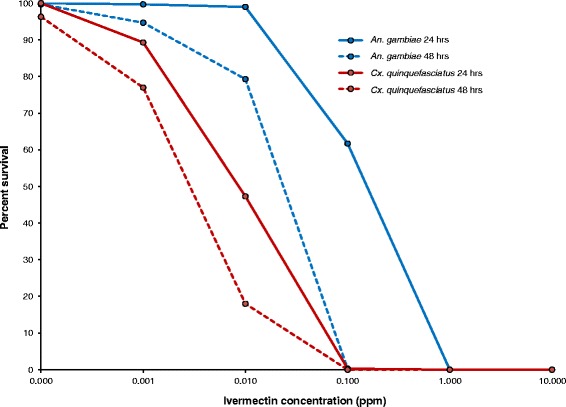
Survival of *An. gambiae* and *Cx. quinquefasciatus* larvae at different ivermectin concentrations and exposure times

Survival was high in the control groups (0.0 ppm ivermectin concentration), being 100 % and 99.6 % for *An. gambiae,* and 100 % and 96.4 % for *Cx. quinquefasciatus,* at 24 and 48 h, respectively. In both species, larval survival declined markedly with increased ivermectin concentration (Fig. 1). For *An. gambiae*, the mean survival in 0.001, 0.01, and 0.1 ppm ivermectin at 24 h was 99.6 % (*P* = 0.3), 99.2 % (*P* = 0.07), and 61.6 % (*P* < 0.001), and at 48 h was 95.2 % (*P* = 0.004), 81.1 % (*P* < 0.001), and 3.2 % (*P* < 0.001) respectively, when compared to the control group. No *An. gambiae* survived for 24 h in 1.0 and 10.0 ppm ivermectin. For *Cx. quinquefasciatus*, the mean survival in 0.001 and 0.1 ppm of ivermectin at 24 h was 89.2 % (*P* = 0.001) and 47.2 % (*P* = 0.001) and at 48 h it was 80.1 % (*P* = 0.001) and 18.7 % (*P* < 0.001), respectively, when compared to the control group. No *Cx. quinquefasciatus* larvae that survived for 24 h in 0.1, 1.0 or 10.0 ppm ivermectin.

Comparison between the species revealed that *Cx. quinquefasciatus* larvae were more susceptible to ivermectin than those of *An. gambiae* (Table 1). At 24 h, mean survival in *An. gambiae* was significantly higher than in *Cx. quinquefasciatus* at ivermectin concentration of 0.001 (*P* = 0.002) and 0.01 (*P* < 0.001) ppm. While ivermectin concentration of 0.1 ppm at 24 h caused 100 % mortality in *Cx. quinquefasciatus,* the same concentration caused only 38.4 % mortality in *An. gambiae*. At 48 h, the same pattern was seen, although mortality was higher in both groups.

## Discussion

Ivermectin is generally considered one of the most beneficial biopharmaceutical drugs for use on a large scale in veterinary and human medicine [1]. Due to its therapeutic effectiveness and broad spectrum of activity in controlling many tropical parasitic diseases, it has been argued that the scope and use of this drug may possibly expand in the near future [25]. However, the broad spectrum of activity has also raised concerns with respect to its impact on non-target organisms in terrestrial and aquatic environments [5]. Of particular importance in mosquito larvae ecology, the bioavailability of ivermectin in aquatic environments is not homogeneous due to its low water solubility and association with sediments. This study assessed the sensitivity of insectary reared *An. gambiae* and *Cx. quinquefasciatus* mosquito larvae (known to feed in different levels of the water strata) to low concentrations of ivermectin under laboratory conditions.

Ivermectin concentrations in soil, groundwater, surface water and animal dung have previously been documented, and have been shown to vary with soil type and route and frequency of application to domestic animals [26]. Non-target organisms have shown variable sensitivity to ivermectin, with the cladoceran *Daphnia magna* being particularly sensitive with 50 % mortality after 48 h of exposure to a concentration of 0.0000057 ppm [27]. Relatively higher ivermectin concentrations of 0.18, 0.0075, 0.78 and 4.8 ppm were found to cause 50 % mortality in non-target Amphipoda, Polychaeta, Gastropoda and Actinopterygii, respectively [28–31]. A number of studies have indicated that photo-degradation on water surface and rapid adsorption of ivermectin to sediments play key roles in determining the bioavailability and environmental fate of ivermectin [32, 33]. Other studies have indicated that benthic microcrustaceans and nematodes generally are more sensitive to ivermectin than non-benthic organisms [27, 34], and this has been argued to be due to strong binding of ivermectin to soil particles thus rendering sediment-dwelling and benthic organisms particularly exposed [35].

In the current study, the survival of both *An. gambiae* and *Cx. quinquefasciatus* larvae declined markedly with increase in ivermectin concentration at both 24 and 48 hours. The study thus confirmed previous observations indicating that ivermectin at low concentrations impairs the survival of larvae of *Cx. quinquefasciatus* [21, 22, 36], and that an ivermectin-related compound ‘spinosad’ (also a macrocyclic lactone) can be effective in controlling both anopheline and culicine mosquito larvae [37–39]. The current study moreover revealed that *Cx. quinquefasciatus* larvae were more susceptible to ivermectin than those of *An. gambiae* at both exposure times. For example, after 24 hours of exposure, an ivermectin concentration of 0.1 ppm caused 100 % larval mortality in *Cx. quinquefasciatus* but only 38.4 % mortality in *An. gambiae.* Our findings thus corroborate with those of Romi et al. [38] who showed that the bioinsecticide spinosad impacted more marked activity against larvae of *Culex* and *Aedes* than against those of *Anophelines*.

Inversely, previous experiments with adult mosquitoes of the same two species showed that blood meals taken from ivermectin treated humans significantly reduced survival of *An. gambiae* but had no effect on *Cx. quinquefasciatus* [10]. The reason for this reduced sensitivity of adult *Cx. quinquefasciatus* to ivermectin in blood meals is a subject for further research. However, the difference in susceptibility to ivermectin in aqueous environments between larvae of the same two species of mosquitoes may in part be associated with the physical properties of ivermectin of low water solubility and rapid adsorption to sediments [15, 16], in combination with the sedimentation of the larval feeds. Thus, it is likely that the larval food particles that sink increase ivermectin bioavailability at the bottom while depleting the same at the water surface. Being surface feeders, *An. gambiae* larvae would be less exposed to ivermectin than the bottom feeding *Cx. quinquefasciatus* larvae. Although it cannot be excluded that *Cx. quinquefasciatus* larvae are inherently more susceptible to ivermectin than *An. gambiae* larvae, it appears likely that the former species is considerably more exposed to the drug due to its bottom feeding habit, and that this may be a major cause for the higher mortality observed for this species. More studies should be carried out in controlled environments to confirm to what extent feeding and dwelling characteristics determine the survival of mosquito larvae in ivermectin contaminated habitats. Field studies should also be undertaken to examine to what extends breeding sites are contaminated in areas with ivermectin mass drug administration and its effect on mosquito larvae.

## Conclusions

Low concentrations of ivermectin in the aquatic environment reduced the survival of larvae of *An. gambiae* and *Cx. quinquefasciatus*, with the effect being more marked in the latter than the former species. As ivermectin has low water solubility and adsorb rapidly to sediments in aquatic environment, this difference in mosquito survivorship may be due to the bottom feeding *Cx. quinquefasciatus* larvae being more heavily exposed to ivermectin than the surface feeding *An. gambiae* larvae. The observed effects on the larvae suggest that ivermectin finding its way to the environment after administration to humans or animals could indirectly affect the vector populations, with different effect on different species, and thereby the transmission of mosquito-borne infections.
